# Performance of Ultrasonography Compared to Conventional Radiography for the Diagnosis of Osteoarthritis in Patients With Knee Pain

**DOI:** 10.3389/fmed.2020.00319

**Published:** 2020-07-03

**Authors:** Martin Brom, Ignacio J. Gandino, Johana B. Zacariaz Hereter, Marina Scolnik, Florencia B. Mollerach, Leandro G. Ferreyra Garrott, Josefina Marin, Santiago O. Ruta, Javier E. Rosa, Ricardo D. García-Mónaco, Enrique R. Soriano

**Affiliations:** ^1^Rheumatology Department, Hospital Italiano de Buenos Aires, Buenos Aires, Argentina; ^2^Radiology Department, Hospital Italiano de Buenos Aires, Buenos Aires, Argentina

**Keywords:** radiography (D011859), ultrasonography (D014463), osteoarthritis (D010003), knee osteoarthritis (D020370), diagnostic imaging (D003952)

## Abstract

**Purpose:** To investigate the performance of ultrasonography (US) for the detection of knee osteoarthritis (OA) in patients suffering from knee pain, compared to conventional radiographs.

**Methods:** Cross-sectional study performed at a university teaching hospital. Consecutive patients complaining of unilateral or bilateral mechanical knee pain who signed an informed consent were included. All patients underwent simultaneously an ultrasonographic and a radiographic evaluation of the knee. Exclusion criteria were age under 18 years, prior diagnosis of knee OA, diagnosis of inflammatory arthritis, history of knee surgery or trauma, severe knee deformities, and corticosteroid injection within the last 2 months. The diagnostic properties of US for the detection of knee OA were evaluated using radiological data as the reference method. Evaluated test properties were sensitivity, specificity, positive predictive value (PPV), negative predictive value (NPV), and the positive and negative likelihood ratio (LR+ and LR–).

**Results:** Three-hundred twenty-two knees (281 patients) were included. Radiographic degenerative changes were present in 56.8% (183) of the evaluated knees. Regarding the diagnostic properties of the US, the presence of either osteophytes or the compromise of the femoral hyaline cartilage had the best sensitivity to detect OA (95%), with a NPV of 92% and a LR– of 0,07, while the combined identification of osteophytes and compromise of the femoral hyaline cartilage had the best specificity (94%), with 94% PPV and a LR+ of 13.

**Conclusion:** US demonstrated an excellent sensitivity with an adequate specificity for the detection of radiographic knee OA.

## Introduction

Osteoarthritis (OA) is the most prevalent joint disorder, with a global age-standardized prevalence of knee OA of 3.8% (1), and it is one of the leading causes of global disability (2). It is characterized by mechanical joint pain and stiffness, and the most frequently affected joints are knees, hips, hands, and spine (3).

Although OA may be diagnosed merely by the presence of typical symptoms and signs in the at-risk group of patients (2, 4, 5), additional testing might be needed to rule out alternative diagnoses and especially, to stratify the degree of joint structural damage. The most frequently used imaging tool is conventional radiography since it is a widely available and cheap method that allows the detection of OA's classical features: marginal osteophytes, joint space narrowing, subchondral sclerosis, and cysts (5–7).

On the other hand, the use of X-Rays in OA has several limitations. Early diagnosis of OA cannot be achieved by this method since X-Rays can only identify late, non-reversible joint damage. Previous studies demonstrated that among patients with knee pain suspicious of OA, only 50% will have radiographic changes of OA (3), and moreover, there is only a moderate association between the degree of knee OA and the level of pain (4, 8) and only about 50% of the patients with knee OA experience pain (9). Pain in knee OA is multifactorial and it is influenced by mechanical, structural, inflammatory, bone-related, neurological and psychological factors (10, 11).

Considering the previously listed limitations of conventional radiography, it is important to have more sensitive tools for the diagnosis and assessment of knee OA. The most sensitive and specific diagnostic tool up to date is Magnetic Resonance Imaging (MRI), since it can evaluate all articular and periarticular structures—including bone marrow, and even biochemical composition of the articular tissues—but its use is limited to research due to the high cost and unavailability of MRI, and its contraindication on certain patients such as those with cardiac pacemakers (12).

Ultrasonography (US) is an attractive tool since, in contrast to conventional radiography, it can evaluate periarticular soft tissue structures and the presence of synovitis, and compared to MRI, it is a safe, inexpensive and less time-consuming method (13). Although US is an operator-dependent method, several studies demonstrated that the use of US in knee OA has good construct validity and moderate to good interobserver reliability (14–17).

In this context, we decided to investigate the properties and performance of US for the detection of knee OA in patients suffering from knee pain, compared to the conventional radiographic study of the knee.

## Patients and Methods

Cross-sectional study performed at a university teaching hospital in Buenos Aires, Argentina. Consecutive patients complaining of unilateral or bilateral mechanical knee pain who signed an informed consent were included. All patients underwent US and X-Ray evaluation of the knee. Exclusion criteria were age under 18 years, prior diagnosis of knee OA, diagnosis of inflammatory arthritis, history of knee surgery or trauma, severe knee deformities, and corticosteroid injection within the last 2 months. All procedures were in accordance with the ethical standards of the institutional research committee, and with the 1964 Helsinki declaration and its later amendments or comparable ethical standards.

US examinations were performed by 2 experienced rheumatologist ultrasonographists (SOR and JER), blinded to clinical and radiological data, using a MyLab 70 machine (Esaote) provided with a multi-frequency linear transducer (4–13 MHz). Patients were not allowed to speak to the ultrasonographist about their clinical condition. A standardized scanning method was adopted in order to evaluate the presence of osteophytes and degenerative femoral hyaline cartilage involvement. Patients were placed in supine position, and knees were evaluated in extension and in 30° flexion. Osteophytes were defined as protrusions at the joint margin seen in two planes. The degenerative femoral hyaline cartilage was defined by the presence of at least two of the following: loss of sharpness of the cartilage margins, loss of homogeneity of the cartilage layer or cartilage thinning (<2 mm) focal or extend to the entire cartilaginous layer.

Weight-bearing anteroposterior and lateral knee radiographs, acquired simultaneously to the US, were read by an experienced rheumatologist, blinded to the clinical and US data, who determined the presence or absence of radiological degenerative changes and classified the severity of knee OA using Kellgren-Lawrence (KL) grading scale (18).

Statistical analysis was performed using STATA V.14.1. Continuous variables were reported as mean and standard deviation (SD), according to the variable distribution. The Chi^2^ Test was used for the comparison of the US features (osteophytes and hyaline cartilage involvement) with the radiographic KL grades, with a *p* < 0.05 considered significant. The diagnostic properties of US for the detection of osteophytes and degenerative cartilage involvement in the knee were evaluated using radiological data as the reference method. Evaluated test properties were sensitivity, specificity, positive predictive value (PPV), negative predictive value (NPV), and the positive and negative likelihood ratio (LR+ and LR–).

## Results

Two-hundred eighty-one patients were included, with a female-to-male ratio of 3:1 and a mean age of 64 years (*SD* 17). A total of 322 knees were evaluated since 41 patients complained of bilateral knee pain.

Table 1 shows the frequency of the US abnormal findings stratified by the radiographic extent of knee damage according to KL grading. Radiographic degenerative changes were present in 56.8% (183) of the evaluated knees, being KL 3 the most frequently observed grade. Regarding the US assessment, the presence of osteophytes or femoral hyaline cartilage involvement was more frequent in those knees with radiographic changes of OA, regardless of the severity according to the KL grading scale (*p* < 0.001 both for hyaline cartilage and osteophytes between all KL grades with radiographic damage and KL0, respectively). The damage of the femoral hyaline cartilage was significantly more frequently found in knees with radiographic knee OA KL 3 and 4 (97 and 100%, respectively; *p* < 0.001 between KL3 and KL4 compared with KL1), while the frequency of osteophytes detected by US was lowest in KL 1 (62%) compared to the other grades (*p* = 0.0067, and 0.0027 vs. KL3 and KL4, respectively). Noteworthy, 24% of the evaluated knees didn't show radiographic degenerative changes (KL 0) but did have ultrasonographic findings of knee OA. On the other hand, none of the patients with radiographic degenerative changes (KL ≥1) had a normal US.

**Table 1:** Frequency of the US abnormal findings according to radiographic features of knee OA.

	**Presence of radiological degenerative changes**, ***n*****: 183**				**Absence of radiological degenerative changes**
	**KL 1, *n*: 34**	**KL 2, *n*: 20**	**KL 3, *n*: 115**	**KL 4, *n*: 14**	**KL 0, *n*: 139**
US femoral hyaline cartilage involvement, % (CI 95%)	77 (64–92)	70 (49–90)	97 (93–99)	100	24 (17–31)
US osteophytes, % (CI 95%)	62 (44–73)	90 (76–100)	85 (79–92)	100	14 (8–20)
US femoral hyaline cartilage involvement or US osteophytes, % (CI 95%)	92 (82–100)	100	95 (91–99)	100	24 (16–31)

*US, ultrasound; OA, osteoarthritis; KL, Kellgren-Lawrence*.

Diagnostic test properties of the US abnormal findings for the detection of knee OA using radiological data as the reference method are shown in Table 2. The presence of osteophytes or the compromise of the femoral hyaline cartilage had the best sensitivity to detect OA (95%), with a NPV of 92% and a LR– of 0.07. The identification of osteophytes and compromise of the femoral hyaline cartilage the best specificity (94%), with 94% PPV and a LR+ of 13.

**Table 2:** Diagnostic test properties of the US abnormal findings for the detection of knee OA using radiological data as the reference method.

	**Sensitivity**	**Specificity**	**PPV**	**NPV**	**LR+**	**LR-**
US femoral hyaline cartilage involvement, % (CI 95%)	90 (86–95)	75 (68–83)	84 (79–89)	85 (78–91)	3.6	0.13
US osteophytes, % (CI 95%)	82 (77–88)	86 (80–92)	89 (84–94)	78 (71–84)	5.85	0.21
US femoral hyaline cartilage involvement or US osteophytes, % (CI 95%)	95 (92–98)	76 (69–83)	85 (80–90)	92 (87–97)	3.96	0.07
US femoral hyaline cartilage and US osteophytes, % (CI 95%)	75 (68–81)	94 (89–97.5)	94 (89–97.6)	74 (67–80)	13	0.27

*US, ultrasonography; PPV, positive predictive value; NPV, negative predictive value; CI, confidence interval; LR+, positive likelihood ratio; LR–, negative likelihood ratio*.

## Discussion

This cross-sectional study shows that US has very good diagnostic properties compared to the standard method, conventional radiography, for the diagnosis of knee OA. The definition and diagnosis of osteoarthritis has changed over time, driven by an increased understanding of the disease and the development of new technologies, which led to the appearance of concepts such as early OA (19). Ultrasonography has growing evidence as an alternative imaging method for the assessment of OA (14). Unlike conventional radiography, US has the ability to visualize articular and periarticular soft tissue structures such as cartilage thickness and soft tissues alterations such as joint effusion, synovitis, Baker's cyst, tendinopathy, bursitis, and meniscal lesions (8, 20–22). On the other hand, and in contrast to MRI, it is a widely available and inexpensive imaging method (13). Additionally, US features such as joint space narrowing and synovitis have been found to better correlate with pain than structural damage (23, 24), and US might be good tool to measure changes in clinical trials (13, 14, 25).

In this context we decided to perform a study to evaluate the diagnostic properties of US for the detection of knee OA in patients who sought medical attention due to knee pain. Although US allows the assessment of many soft tissue structures, the ultrasonographic evaluation of the knees in this cohort was limited to the presence of osteophytes and hyaline cartilage involvement since these are the only joint changes that can be observed both by US and conventional radiography, and therefore, these are the features that allow the comparison of the diagnostic properties of these 2 imaging methods.

Several studies have shown US to be reliable and valid for the evaluation of cartilage pathology in OA, and especially for large joints such as the knee (26–29), and US has demonstrated strong criterion validity with cartilage histology (25). The knee's hyaline cartilage has a normal thickness of 2 mm, and superficial regions can easily be evaluated by US (26, 27).

In this cohort, 56.8% of the patients with knee pain had radiological degenerative changes, and in concordance with previous studies (20, 30), KL 3 was the most frequently identified group. Interestingly, 24% of the patients without radiographic degenerative changes presented ultrasonographic findings of OA. Whether this represents a false positive result of US or the diagnosis of early OA can only be elucidated by following these knees in time, and cannot be answer by this study.

Previous studies demonstrated that US is as sensitive as MRI and more sensitive than conventional radiography for the detection of osteophytes (25, 26, 31–34), and showed a high intra and inter-reader agreement (26–28, 35). In this cohort, ultrasonography had a sensitivity of 95% (CI95% 92–98) for the detection of hyaline cartilage involvement or osteophytes, and a specificity of 86% (CI95% 80–92) for the identification of osteophytes, compared to conventional radiography. Using both features, as expected, increased specificity but decreased sensitivity (Table 2). On the other hand, if we had used US as gold standard, conventional radiography would have shown sensitivity of 83% and specificity of 93%.

There are some limitations that need mentioning. First, there are limitations that are inherent to the method: The visualization of the cartilage may be hindered by the acoustic window, which depends on the patient's joint anatomy, and US is an operator-dependent method (15). Second, inter and intraobserver agreement was not evaluated, which might affect reliability. Nevertheless, the participating ultrasonographists are part of the same group and share the same concepts on acquisition and reading of images. In addition, they have demonstrated a very good level of agreement (85%) in a previous studies (36). Third, although we are aware that looking for a correlation between the images and pain or clinical characteristics such as Body Mass Index (BMI), etc. would enrich the study, since the objective was the comparison of the diagnostic properties of US and radiographs and patients did not undergo an *ad hoc* physical examination, this was not possible. Finally, since this is a cross-sectional study, we cannot know if the changes found by US in knees with normal radiographs (KL 0) are false positive results or if it is an early diagnosis of OA.

On the other hand, this study has several strengths. This is a large cohort of patients, who were included consecutively and unselected. All patients were evaluated by 2 rheumatologists expert in ultrasonography, who were blinded to the KL grading. Likewise, the radiographic evaluators were expert rheumatologists, blinded to the US findings.

In conclusion, US demonstrated an excellent sensitivity with an adequate specificity for the detection of radiographic knee OA. The identification by US of femoral hyaline cartilage involvement or osteophytes showed the best sensitivity while the presence of both osteophytes and femoral hyaline cartilage showed the best specificity. We believe that ultrasonography is a valid method for the evaluation of patients with knee pain when OA is suspected. Its capability to identify early knee OA in patients with non-radiographic knee OA (KL 0) should be evaluated using as a more sensitive method, such as MRI, as the comparator group.

## Data Availability Statement

The raw data supporting the conclusions of this article will be made available by the authors, without undue reservation.

## Ethics Statement

The studies involving human participants were reviewed and approved by Hospital Italiano de Buenos Aires. The patients/participants provided their written informed consent to participate in this study.

## Author's Note

This study was previously published as an abstract after being presented at an ACR meeting (37) (https://acrabstracts.org/abstract/radiographic-knee-osteoarthritis-in-patients-complaining-of-knee-pain-ultrasound-features/) and a PANLAR meeting (38) (https://journals.lww.com/jclinrheum/Fulltext/2018/04001/Abstracts_20th_PANLAR_Meeting__Buenos_Aires,0.1.aspx).

## Author Contributions

MB, IG, JZ, MS, FM, LF, JM, SR, JR, RG-M, and ES meet the ICMJE 4 criteria and the journal authorship criteria, and the final manuscript has been read and approved by all the authors.

## Conflict of Interest

The authors declare that the research was conducted in the absence of any commercial or financial relationships that could be construed as a potential conflict of interest.
